# Molecular Identification and Phylogenetic Placement of *Rosa arabica* Crép. (Rosaceae), a Critically Endangered Plant Species

**DOI:** 10.3390/life10120335

**Published:** 2020-12-09

**Authors:** Ahmed EL-Banhawy, Carmen Acedo, Sameer Qari, Ahmed Elkordy

**Affiliations:** 1 Botany Department, Faculty of Science, Suez Canal University, Ismailia 41522, Egypt; 2 Biodiversity and Environment Management Department, Faculty of Biological and Environmental Sciences, University of León, 24071 León, Spain; c.acedo@unileon.es (C.A.); aelkordy@science.sohag.edu.eg (A.E.); 3 Biology Department, Aljumum University College, Umm Al-Qura University, Makkah 21421, Saudi Arabia; 4 Botany and Microbiology Department, Faculty of Science, Sohag University, Sohag 82524, Egypt

**Keywords:** biosystematics, *Caninae*, *Rosa arabica*, phylogeny, endemic, Saint Catherine, Sinai, Egypt

## Abstract

The Egyptian narrowly endemic and critically endangered plant species *Rosa arabica* Crép. was studied employing a taxonomic and molecular approach. Morphological investigations, distance analysis, and phylogenetic reconstruction revealed that *R. arabica* is a distinct species with great affinity to *R. canina* and differentiated from *R. rubiginosa*. Molecular identification based on the sequences of multiple markers single or in combination ITS, *mat*K, *rbc*L, and *trn*L-F succeeded in identifying *R. arabica* at genus and species levels. We evaluated the potential of each marker and a combination of the nuclear ITS -Internal Transcribed Spacer- with one of the plastid markers, *mat*K, *rbc*L, or *trn*L-F, to accurately identify *Rosa* species. All of them were successful in identifying *R. arabica*. Classification based on DNA sequences shows that *R. arabica* is placed within section *Caninae* in a clade comprising *R. canina* and *R. rubiginosa*. Moreover, *R. arabica* is closely related to other European *Rosa* species. In conclusion, our results indicate that the four DNA markers can provide species resolution in the context of the genus *Rosa* and relatives, aiming to characterize morphology and genetic diversity in the ecological and economically important genus Rosa.

## 1. Introduction

The family Rosaceae is a large sub-cosmopolitan family located mainly in the temperate and warm areas of the Northern Hemisphere [1]. Heywood [2] stated that the family consists of 122 genera and 3370 species. Christenhusz et al. [3], reported 54–75 (90) genera and 2950 species. The genus *Rosa* L. comprises approximately 200 species [4]. Recently, ref. [5] updated those numbers as 5819 species in 109 genera and 310 species in the Genus *Rosa*.

Conventional taxonomy by Wissemann [6] divides the genus into four subgenera, three of which are monotypic or contain two species (*Hulthemia* Dumort. [7], *Platyrhodon* (Hurst) Rehder [8], and *Hesperhodos* Cockerell [9]) and subgenus *Rosa* L. with 11 sections [10]. In 2005, ref. [11] stated that all the subgenera of *Rosa* are “best treated as sections, and not as separate genera”. They formally transferred all subgenera to sections. This was later supported by [12,13,14].

Hybridization is common within the genus *Rosa*. Hybrids contribute to the diversity of the genus, increasing the difficulty in reconstructing the species relationships based on morphology. Numerous researches investigated the phylogeny of the genus *Rosa*, most of which suggested that the divisions of most subgenera and sections based on morphology were artificial [12,15,16,17].

The Rosaceae family is represented in Egypt by seven taxa belonging to six genera, namely, *Crataegus azarolus* L., *Crataegus* × *sinaica* Boiss., *Cotoneaster orbicularis* Schltall., *Potentilla supina* L., *Rosa arabica* Crép., *Rubus sanctus* Schreb., and *Sanguisorba minor* Scop. Of these taxa, only *Potentilla supina* is ubiquitous, while the others are either of rare or of sporadic occurrence [18,19,20].

The genus *Rosa* is represented in Egypt by only one species, i.e., *R. arabica* Crép. Its vernacular name is Sant Catherine wild rose, or “Ward Barre”. This species is endemic and is on the red list of the world’s most threatened species; it has a narrow distribution restricted to Mount Catherine, South Sinai, Egypt [21].

*Rosa rubiginosa* is a native rose species to Europe and Northern Asia [22]. *R. arabica* was proposed by Crépin, who classified *R. arabica* as an Asian variety of the European *R. rubiginosa* L. [23]. *R. arabica* and *R*. *rubiginosa* were treated as closely related on the basis of their morphology, as well as the many floras, monographs, and electronic sites treated *R. arabica* among the synonyms of *R. rubiginosa* [6,8,23,24]. According to The Plant List database (TPL) [25], the name *R. arabica* is reported as an unresolved name, i.e., not established as either accepted or a synonym. The Weeds of Australia website [26] reports both *R. arabica* and *R. eglanteria* as synonyms of *R. rubiginosa*.

Prior to this study, no phylogenetic study of *R. arabica* was undertaken. The phylogenetic analyses of the genus *Rosa* did not include samples of this species [12,15,16,17].

The origin of endemism is an important evolutionary and taxonomic question. Is *R. arabica* a distinct species? Is it an infraspecific taxon of *R. rubiginosa*? What precisely are its origin, phylogenetic position, and route of evolution? The current study uses classical and cutting-edge taxonomic tools to answer these questions.

## 2. Materials and Methods

### 2.1. Plant Collection

The present study was based on fresh and dried herbarium materials. The fresh materials were collected from Mount Catherine (2629 m), Saint Catherine, South Sinai, Egypt. Herbarium materials were obtained from Egyptian herbaria: Suez Canal University (SCUI, Ismailia, Egypt), Sohag University (SHG, Sohag, Egypt), and Cairo University herbarium (CAI, Cairo, Egypt). The herbarium materials for non-Egyptian species were obtained from the Florida Museum of Natural History, University of Florida Herbarium (FLAS), Gainesville, FL, USA. Herbarium acronyms follow The Index Herbariorum [27].

### 2.2. Selected Specimens Examined

The following specimens were examined: *Rosa arabica*, Egypt, Southern Sinai, Wadi El-Arbaeen, Kahf El-Ghola: 28°32′49″ north (N), 33°56′93″ east (E), 1 May 2016, Ahmed EL-Banhawy & Ahmed Elkordy AEB#301, (SCUI s.n.); Wadi El-Arbaeen, Kahf El-Ghola, 18 June 2005, K. Abdelkhalik, (SHG s.n.); Wadi El-Arbaeen, (Encl. 3): 28°32′56.9″ N, 33°56′56.2″ E, Alt. 1650 m, 2 April 2004, K. Shaltout et al., (Herbarium of Elsalam Botanical Garden, Sharm Elsheikh, s.n.); Saint Catherine, April 1940, M. Hassib (CAI); *Rosa eglanteria* L. USA, Pennsylvania: 1.2 mi. E–NE of Pricetown, Berks County, dry soil in the field, W.C. Brumbach, 4283, 15 June 1950 (FLAS); New York: old field on South Hill near Ithaca, Tompkins Co11, October 1940, E.S. Ford #638 (FLAS); North Carolina: Orange County, University of North Carolina campus, cultivated, Chapel Hill, 9 July 1957, Max Newbery 236 (FALS); *Rosa rubiginosa* L. USA, Oregon: Clackamas County, one mile south of Canby, in open pasture, 12 June 1943 R. H. Belton,1943. (FLAS); Pennsylvania: Berks County, 1.2 mi, S.W. of Weavertown, dry soil along the highway, W. C. Brumbach 3745, 19 June 1944 (FLAS).

### 2.3. Systematic Treatment

#### Identification and Nomenclature

**Figure 1:**
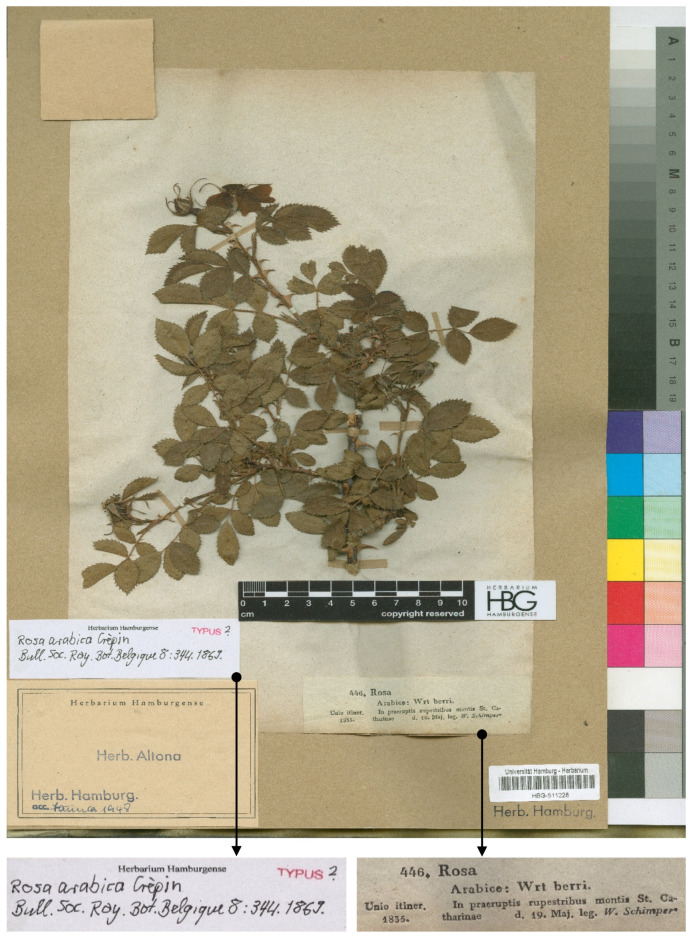
Type of *R. arabica*, collected by W. Schimper from Saint Catharine, South Sinai, Egypt, 19 May 1835, from the original collection, conserved in the Herbarium Hamburgense (HBG 511228). Available at Herbarium Hamburgense. Reproduced with permission.

Examined specimens were identified according to the latest available literature, [18,19,20,22]. An image of several original specimens collected by W. Schimper in 1835 from Saint Catharine, South Sinai, Egypt, was downloaded from the internet repository of the Herbarium Hamburgense (Figure 1).

To assess the traits of the original specimen *R. arabica*, we studied the specimens collected in the *loco classic*. There is no certainty about the type specimen of this species due to the presence of several W. Schimper specimens, and nobody has assigned the type.

A survey of international floras, as well as 10 online databases, was conducted to evaluate the status of *R. arabica* nomenclature (Table 1).

### 2.4. DNA Extraction and PCR Amplification

Fresh leaf materials used for molecular analyses were collected and preserved in silica gel. DNA was extracted using the cetyltrimethylammonium bromide (CTAB) protocol with some modifications [28]. The PCR amplification was performed in 15 µL volume for ITS -Internal Transcribed Spacer-, *mat*K, *rbcL,* and *trn*L-F containing 5 U/µL Taq DNA polymerase with 25 µM MgCl_2_, 10 µM dNTPs, and 10 µM of each primer. Amplifications were conducted using an Applied Biosystems^®^-Veriti™ 96-well thermal cycler (Thermo Fisher Scientific-Fisher Scientific AS-Postboks 114, Smestad-0309 Oslo–Norway). The thermal cycling program for amplification of the ITS region was as follows: 95 °C for 2 min, 34 cycles of 95 °C for 45 s, 58 °C for 45 s, and 72 °C for 90 s, and a final extension at 72 °C for 5 min; that for the *mat*K region was as follows: 95 °C for 3 min, 40 cycles of 94 °C for 30 s, 49 °C for 1 min, and 72 °C for 1 min, and a final extension at 72 °C for 10 min; that for the *rbc*L region was as follows: 95 °C for 6 min, 30 cycles of 95 °C for 45 s, 48 °C for 45 s, and 72 °C for 90 s, and a final extension at 72 °C for 6 min; that for the *trn*L-F region was as follows: 95 °C for 5 min, 15 cycles of 95 °C for 45 s and 60 °C for 1 min, with an extension at 72 °C for 2 min, followed by 20 cycles of 95 °C for 45 s and 54 °C for 1 min, and a final extension at 72 °C for 2 min. The primers used in this study are shown in Table 2.

### 2.5. DNA Sequencing

PCR products were purified with ExoSAP-IT (USB Corporation, Cleveland, OH, USA) according to manufacturer recommendations. PCR products were sent to Macrogen Spain for direct sequencing in both directions with an ABI 3730XL Genetic Analyzer (Life Technologies Corporation, Carlsbad, CA, USA).

These novel DNA sequences of *R. arabica* were deposited in the GenBank under the following accession numbers: ITS, MT358870; *mat*K, MT416573; *rbc*L, MT415957; *trn*L-F, MT427590.

### 2.6. Molecular Data Analysis

#### 2.6.1. Molecular Identification

There is no general agreement for a single method that supports species discrimination using DNA sequence data. During this study, molecular identification and phylogenetic analysis were implemented using multiple approaches [43,44].

#### 2.6.2. BLAST (Basic Local Alignment Search Tool) and Reference Datasets

A total of four markers (one nuclear and three plastid) were selected for this study; 39 ITS, 26 *matK*, 24 *rbc*L, and 16 *trn*L-F sequences of *Rosa* taxa were used in our study. Verified representative sequences of each taxon were tentatively identified using the BLASTN algorithm available on the NCBI –National Center for Biotechnology Information website. Additional sequences were obtained and included in the datasets. GenBank accession numbers and similarity matching percentage are presented in the Supplementary Materials (Table S1).

A reference sequence dataset was constructed, which consisted of sequences matching 92–99% in sequence similarity [45]. For the newly generated sequences, forward and reverse reads were assembled and edited into contigs in GENEIOUS^®^ v.R9 (Biomatters Ltd., Berkeley, CA, USA, 94709-1405, https://www.geneious.com) using a personal license (C.A.). Four data matrices were constructed: ITS, *mat*K, *rbc*L, and *trn*L-F. The ingroup was selected to cover most of the major sections in the genus *Rosa*. Datasets of each marker were initially aligned using ClustalW [46] or MAFFT algorithms [47], implemented in Geneious, using default alignment parameters.

#### 2.6.3. Tree-Based Analysis

Analyses were run on the CIPRES portal [48]. The aligned DNA sequences for three chloroplast DNA (cpDNA) and one nuclear DNA (nrDNA) were used to construct four single markers and three combined datasets. The optimal nucleotide substitution model was estimated using MrModeltest [49] and executed in MrBayes blocks. Monte Carlo Markov chain (MCMC) was conducted using MrBayes 3.0b4 [50]. Four heated MCMC chains were run over 10 million generations, using general time reversible (GTR) plus gamma distribution substitution rates, random seed trees, and the default starting value for the nucleotide substitution model. Trees were sampled every 1000 generations, resulting in 20,001 trees. The first 25% “burn-in” trees were deleted from the analysis. A 50% majority role consensus tree was constructed to get the posterior probabilities (PP). For each analysis, two independent runs were executed using initial parameters. Posteriori probabilities >0.5 at a given branch were considered strong support for the existence of that branch [44,51,52].

## 3. Results

### 3.1. Taxonomic Identity of Rosa arabica

Shrubs and shoots were 0.5–1.5 m tall, erect or scrambling, terete, sparsely prickly, usually curved or hooked, with or without a stout base. Branchlets were dark brownish red, 5.0–10 mm in diameter, pubescent or glabrate. Prickles were dark brownish, stout, falcate up to 1.5 cm. Leaves were alternate, odd-pinnately compound, including petioles 0.5–1.2 × 0.2–0.3 cm. Stipules were 4–10 × 2–4 mm, apex acute, margin doubly serrate. Leaflets were 5–7 in number, broadly elliptic to elliptic obovate, 10–30 × 6–18 mm, abaxially green, sparsely pubescent with glandular trichome, adaxially green, glabrous, sparsely pubescent with glandular trichome, or slightly setose along midrib, apex acute, margin doubly glandular–serrate, base rounded. Flowers were solitary, 3.5–4.5 cm in diameter, pedicel ca. 10 mm long, setose–glandular trichome, as long as or longer than the fruit, bracts lanceolate, 8.0–12 × 2.0–5.0 mm, margins stipitate-glandular, glabrous, and eglandular surfaces. The hypanthium was globose, bright red, setose to sparsely glandular trichome. Sepals were five in number, reddish green, broadly lanceolate, 9–21 × 2.5–4.0 mm, abaxially setose–glandular trichome, adaxially densely tomentose, attenuate apex, margin lobed, pinnatifid, recurved, and persistent after anthesis. Petals were five in number, free, pink to deep rose, fragrant, obovate, 9.0–20 × 6.0–10 mm, entire margin, emarginate apex, and cuneate base. Stamens were numerous filaments, 3.0–5.0 mm. Styles were filiform, 4.0–6.0 mm long, exerted, slightly longer than stamens. Fruits were hip, usually ripe with bright red color, globose, 10–21 × 8.0–19 mm, sparsely setose glandular trichome, shiny. Achenes were ovoid to sub elliptic, trigonous, 3.5–4.0 × 2.5–3.0 mm, ventral side roof like with a longitudinal suture, acute apex, and mostly truncate base. Main differences with *R. rubiginosa* are listed in Table 3.

Flowering occurred from May to June, while fruiting occurred from June to July.

Its distribution is restricted to Egypt, Southern Sinai, Endemic.

Type: *Rosa arabica* Crépin. Herbarium Hamburgense. Bull. Soc. Roy. Bot. Belgique 8(2):344. 1869. Egypt, South Sinai, Saint Catharine, 1835, W. Schimper s.n. (HBG 511228) [53] (Figure 1).

To determine the current state of the name of *R. arabica*, we surveyed 10 electronic databases (Table 1). The results showed that there is some ambiguity in nomenclature and taxonomic status. The Plant List Database (TPL) retrieved the name *R. arabica* without its related taxonomic or nomenclature status. The World Flora Online (WFO) database considered *R. arabica* as an ambiguous name, while the Catalog of Life database showed *R. arabica* as an accepted name. Moreover, the Weeds of Australia retrieved *R. arabica* as a synonym for *R. rubiginosa,* while the name *R. arabica* is not recognized by the Tropicos database. The International Plant Name Index (IPNI), Plant of the World Online (PoWo), Global Biodiversity Information Facility (GBIF), Integrated Digitized Biocollections (IDigBio), and Open Tree of Life databases accepted the name *R. arabica* as a distinct species.

### 3.2. Molecular Identification Approach

For the first time, DNA sequences of ITS, *matK*, *rbc*L, and *trn*L-F markers were generated and used for molecular identification of *R. arabica*. Generally, the single markers ITS, *mat*K, *rbc*L, and *trn*L-F succeeded in identifying the query sequence at the genus and species level and provided support to discriminate the species *R. arabica* (Table S2). The ITS and *mat*K markers displayed the highest-level discriminatory power.

### 3.3. Phylogenetic Relationship

Bayesian inference (BI) of the four single markers ITS, *mat*K, *rbc*L, and *trn*L-F, combined datasets ITS + *mat*K and ITS + *rbc*L, and a concatenated dataset of all four markers was conducted.

**Figure 2:**
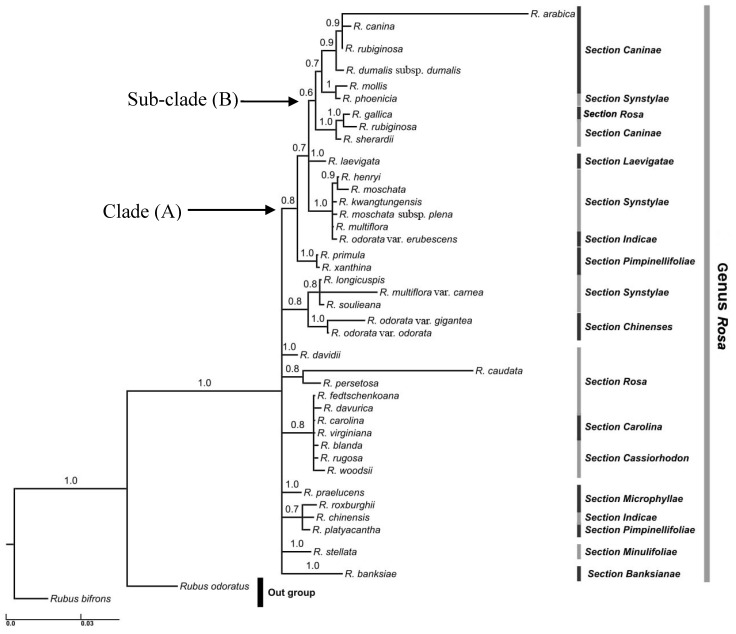
Bayesian phylogenetic tree of ITS marker showing the relationships between *R. arabica* and representatives of most *Rosa* sections. Numbers above branches are posterior probability (PP) values >0.6. Accessions numbers of sequences retrieved from GenBank are detailed in Table S1 (Supplementary Materials). Clade A and Sub-Clade B defined in the text.

**Figure 3:**
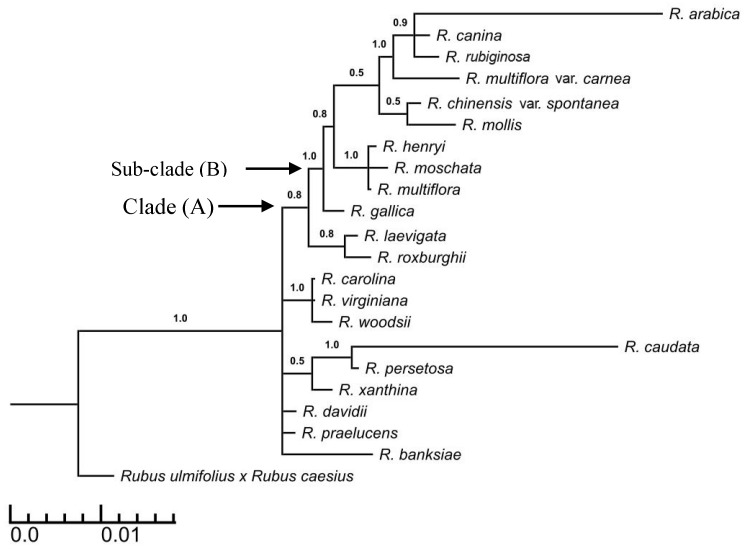
Bayesian phylogenetic tree of combined datasets of ITS + *mat*k markers, showing the relationships between *R. arabica* and representatives of most *Rosa* sections and outgroups. Numbers above branches are PP values >0.5. Accessions numbers of sequences retrieved from GenBank are detailed in Table S1 (Supplementary Materials). Clade A and Sub-Clade B defined in the text.

The dataset of the single marker ITS (Figure 2), and the combined ITS + *mat*K (Figure 3), retrieved two congruent phylogenetic trees. The ITS phylogenetic tree comprised 39 *Rosa* species, as well as two outgroup taxa: *Rubus bifrons* and *Rubus odoratus*. Despite some polytomies, a moderately resolved clade A consisted of 18 taxa. In clade A, *R. primula* and *R. xanthine* were sister groups. The subclade B “posterior probabilities (PP) = 0.6” comprised nine *Rosa* species including *R. arabica* (Figure 2).

All taxa under investigation belonged to the genus *Rosa*. The current study shows genus *Rosa* subdivided into 12 sections. Clade A includes six sections: section *Caninae* “eight taxa” section *Synstaylae* “six taxa” section *Pimpinellifoliae* “two taxa” and two more sections with a single taxon, section *Rosa*, and section *Laevigatae* (Figure 2).

The phylogenetic tree obtained from the combined dataset comprised 21 *Rosa* species and *Rubus ulmifolius* × *R. caesius* as an outgroup. Clade A was composed of 12 *Rosa* species. *R. laevigata* and *R. roxburghii* were sisters to subclade B. Subclade B (PP = 1) comprised 10 *Rosa* species including *R. arabica*. Most of the main clades were moderately to highly supported (0.7–1) (Figure 3).

Nei’s [54] distance analysis (Table S2) applied to the ITS marker between *R. arabica* and *R. rubiginosa* and *R. canina* was 96.25 and 81.93. In *mat*K, the distance between *R. arabica* and *R. canina* and *R. rubiginosa* was 99.70 and 99.79. In *rbc*L, the distance between *R. arabica* and *R. canina* and *R. rubiginosa* was (97.45 and 97.97). In *trn*L-F, the divergence between *R. arabica* and *R. canina* and *R. rubiginosa* was 48.78 and 48.84.

## 4. Discussion

According to the IUCN – International Union for Conservation of Nature—criteria of plant conservation, the Saint Catherine wild rose *R. arabica* is considered to be one of the most critically endangered plant species in Egypt [21], which justified our interest in its phylogenetic placement.

Tomljenović and Pejić [55] reviewed the taxonomy of the genus *Rosa*. They discussed the efforts to classify and systematize roses from the 16th century until recently. They determined that species discrimination based on morphological characterization was very challenging over the last three centuries due to (1) few described species, (2) a low number of apparent morphological differences between closely related species, and (3) extensive hybridization and polyploidy. Tomljenović and Pejić [55] also suggested that modern tools of classification, such as molecular markers and phylogenetic analyses, as well as traditional morphological methods, would be of great help in clarifying the phylogenetic relationships within the genus *Rosa*.

The inability to retrieve the correct information for a particular plant species nomenclature status using taxonomic or biological databases might arise from the accumulation of outdated information (e.g., TPL has been static since 2013 but was used as the starting point for the Taxonomic Backbone of the WFO). We recommend that plant databases should be curated regularly with the findings of recent taxonomic and ecological studies incorporated. This is especially necessary where the plants concerned are rare and threatened, as is the case with *R. arabica*.

Although there is no certainty about the type specimen of the name *R. arabica* Crép., it was possible to study several specimens cited by [23] that are part of the original material; however, a more detailed and specific study is needed to confirm typification of the name.

*Rosa arabica* has never been the subject of extensive taxonomic or phylogenetic investigation. Even though the identification and nomenclature of *R. arabica* seem to be straight forward, they have represented a challenge even for some experts. Although *R. arabica* and *R*. *rubiginosa* have been treated as closely related based on their morphology, and many floras and databases treated *R. arabica* as a synonym of *R. rubiginosa* [6,7,8,23,24], several critical distinctive morphological traits differentiate *R. arabica* and *R. rubiginosa*. The most important diagnostic features for *R. arabica* are stout prickles, falcate up to 1.5 cm (falcate or curved in *R. rubiginosa*), leaflets shaped broadly elliptic to obovate (suborbicular in *R. rubiginosa*), the texture of the leaflet upper surface being glabrous, sparsely glandular, or slightly setose along midrib (glabrous or pubescent in *R. rubiginosa*), and the hypanthium texture being setose to sparsely glandular in *R. arabica* (glabrous or glandular–hispid in *R. rubiginosa*), Table 3.

By applying tree-based analysis, the query sequence was assigned to a species if clustered with sequences from their correct taxon with strong support value [44]. The current study recommends the use of ITS, *mat*K, *rbc*L, and *trn*L-F as molecular marker candidates for identification of *R. arabica*.

Sectional classification of the genus *Rosa* was partially retrieved using analyses of a single dataset of DNA sequences of the nuclear marker ITS, as well as a combined dataset of DNA sequences of the chloroplast marker (ITS + *mat*K). The genus *Rosa* is composed of 12 sections: *Caninae*, *Synstaylae*, *Rosa*, *Chinensis*, *Laevigatae Pimpinellifoliae, Carolina*, *Cassiorhodon*, *Microphyllae*, *Indica*, *Minulifoliae*, and *Banksianae* (Figure 2 and Figure 3). Although the sectional classification of the genus *Rosa* is beyond the scope of the current study, the sectional classification is congruent with previous studies [16]. The present study aimed to place *R. arabica* within its related section within the genus *Rosa* (*s.l*.). According to our Bayesian analysis of the DNA sequences of ITS and ITS + *mat*K, *R. arabica* was placed within section *Caninae* (Figure 2 and Figure 3). Morphologically, section *Caninae* is characterized by pink or white flowers. *R. arabica* exhibits a range of intermediate morphological characters between *R. canina* and *R. rubiginosa*, especially in terms of the petal color; *R. arabica* shows pale to deep rose petals [56].

Distance analysis of DNA sequences represents another excellent tool to compare closely related species. Ussery [57] recommends using multi-locus data distance analysis instead of a single locus. In the current study, we carried out distance analysis using the ITS, *mat*K, *rbc*L, and *trn*L-F data sequences to compare *R. arabica* and its closely related species within the section *Caninae*. The distance analyses indicated that *R. arabica* is much more closely related to those two species (Table S2). All members of section *Caninae* are distributed in Europe, North America, Asia, and North Africa [6].

According to [58], the Egyptian flora is a mix between South Mediterranean and East Asia. The Sinai Peninsula is the contact point between those two geographical routes. Thus, *R. arabica* might be a hybrid and, after having been isolated in its minimal distribution range, became a new species.

## 5. Conclusions

The result of our taxonomical and molecular study confirmed the identity of *R. arabica*. A better taxonomy and molecular distance comparison of this *Rosa* species group support the distinct identity of the Red-Listed *R. arabica*. The phylogenetic results allow redefining its position in the genus *Rosa* and its close relatives, as well as its accurate identification using the identity of molecular markers. The results show that *R. arabica* is a sister of *R. canina* and a relative of *R. rubiginosa* belonging to section *Caninae*. The use of two molecular markers (ITS and *mat*K) can help provide insights into *Rosa* species-level taxonomy, and this may be essential in identifying the correct taxonomy or new species. This will be useful especially where morphology-based identification is difficult. The use of a molecular identification approach for *Rosa* revealed more accurate identification than classical morphologically based taxonomy. A better understanding of the taxonomy and the phylogenetic relationships in *Rosa* is fundamental toward the proposal of conservation strategies. We show that the use of the selected molecular markers represents a powerful tool in cases where correct identification is essential, e.g., for the recognition of critically endangered species that must be protected.

## Figures and Tables

**Table 1:** Nomenclature status and resolution of *Rosa arabica* according to modern biological databases.

Query	Retrieved Name	Name Resolution	Synonym	Taxonomic Status	Database /Flora
*R. arabica*	*R. arabica* Crép.	Accepted	None	Species	IDigBio
	*R. arabica* Crép.	Accepted	*R. rubiginosa*	Species	Open Tree of Life
	*R. arabica* Crép.	Accepted	None	Species	IPNI
	*R. arabica* (Crép. ex Boiss.) Déségl.	Accepted	None	Species	PoWo
	*R. arabica* Crép.	Accepted	*R. rubiginosa* var. *arabica* (Crépin) Boiss.	Species	Catalog of Life
	*R. arabica*	Synonym	*R. arvensis* Huds.	Species	GBIF
	*R. rubiginosa* L.	Synonym	*R. arabica* Crép. *R. eglanteria* L.	Species	Weeds of Australia
	*R. arabica* (Crép. ex Boiss.) Déségl.	Not recognized	Not recognized	Not recognized	Tropicos
	*R. arabica* Crép.	Unresolved	None	Unresolved	TPL
	*R. arabica* (Crép. ex Boiss.) Déségl.	Unresolved	None	Unresolved	
	*R. arabica* (Crép.)	Ambiguous	None	Ambiguous	WFO

TPL: The Plant List [29]; Tropicos: the online, nomenclatural database of the Missouri Botanical Garden [30]; GBIF: Global Biodiversity Information Facility [31]; IDigBio: Integrated Digitized Biocollections [32]; IPNI: International Plant Name Index [33]; PoWo: Plant of the World Online [34]; Open Tree of Life [35]; Catalog of Life [36]; Weeds of Australia [26]; WFO: World Flora Online [37].

**Table 2:** DNA primer sequences used in molecular analysis. F, forward; R, reverse.

Region	Primer F/R	Primer Sequence (5′–3′)	Reference
ITS	KRC	GCACGCGCGCTACACTGA	[38]
	AB102	TAGAATTCCCCGGTTCGCTCGCCGTTAC	[39]
*mat*K	K1R	ACCCAGTCCATCTGGAAATCTTGGTTC	[40]
	K3F	CGTACAGTACTTTTGTGTTTACGAG	
*rbc*L	*rbc*L a-F	ATGTCACCACAAACAGAGACTAAAGC	[41]
	*rbc*L a-R	GTAAAATCAAGTCCACCRCG	
	*trn*L^(UAG)^	CTGCTTCCTAAGAGCAGCGT	
*trn*T-*trn*L	*trn*-b	TCTACCGATTTCGCCATATC	[42]
	*trn*-a	CATTACAAATGCGATGCTCT	
*trn*L (intron)	*trn*-d	GGGGATAGAGGGACTTGAAC	
	*trn*-c	CGAAATCGGTAGACGCTACG	

**Table 3:** List of morphological characters for *R. arabica* and *R. rubiginosa*.

Characters	Taxa	
	*R. arabica* Crép.	*R. rubiginosa* L.
Habitus	Erect or scrambling	Erect
Life form	Shrub	Shrub
Plant height	0.5–1.5 m	1.5–2 m
Prickles	Stout, falcate up to 1.5 cm	Falcate or curved, sometimes mixed with acicles and glandular setae
Leaflets shape	Broadly elliptic obovate	Suborbicular to broadly ovate to obovate
Leaflet No.	5–7	5–7
Leaflets length	10–30 mm	10–25 mm
Leaflets width	6–18 mm	8–15 mm
Leaflets margin	Doubly serrate	Compound–serrate
Leaflet base	Rounded	Rounded
Leaflet upper surface	Glabrous, sparsely glandular, or slightly setose along midtrip	Glabrous or pubescent
Leaflet lower surface	Sparsely glandular	Pubescent or densely glandular–viscid
Pedicels length	10 mm	10–15 mm
Pedicels surface	Setose–glandular	Densely stipitate or setose–glandular
Sepals nature	Recurved, persistent after anthesis	Erect and persistent after anthesis
Sepals dorsal surface	Setose–glandular	Glandular
Sepals ventral surface	Densely tomentose	Glandular
Hypanthium Shape	Globose	Sub globose, ovoid or ellipsoid
Hypanthium surface	Setose–sparsely glandular	Glabrous or glandular–hispid
Hypanthium color	Bright red	Bright red

## Supplementary Materials

The following are available online at https://www.mdpi.com/2075-1729/10/12/335/s1, Table S1. BLAST results for each marker (single locus) of tested samples. Database search match for similarities and phylogenetic relationships of ITS, *mat*K, *rbc*L, and *trn*L-F sequences; Table S2. Matrix of pairwise divergences of ITS, *mat*K, *rbc*L, and *trn*L-F for all samples studied.
