# Supplementary material for: Molecular Identification and Phylogenetic Placement of Rosa arabica Crép. (Rosaceae), a Critically Endangered Plant Species
Source: Life (Basel). 2020 Dec 9;10(12):335. doi: 10.3390/life10120335 (PMC7763824; doi:10.3390/life10120335)
Supplement: Supplementary file 1 [file life-10-00335-s001.pdf]

life

MDPI

Supplementary materials of Molecular Identification and Phylogenetic Placement of *Rosa arabica* Crép. (Rosaceae), a Critically Endangered Plant Species

Table S1. BLAST results for each barcode (single locus) of tested samples. Database search match for similarities and phylogenetic relationships of ITS, *mat*K, *rbc*L, and *trn*L-F sequences.

Marker	Retrieved taxa	Accession No.	Similarity Matching %
ITS	*Rosa arabica*	MT358870	-
	Rosa canina	FM164424.1	100.00
	Rosa eglanteria	FM164946.1	98.00
	Rosa dumalis subsp. *Dumalis*	*FM164950.1*	*93.48*
	Rosa mollis	FJ948761.1	98.00
	Rosa phoenicia	AB043829.1	90.48
	Rosa gallica	AB043824.1	95.0
	Rosa rubiginosa	AJ631885.1	97.49
	Rosa sherardii	FJ947108.1	96.00
	Rosa laevigata	KP093154.1	91.52
	Rosa henryi	KP0931151.1	91.52
	Rosa moschata	AB043004.1	90.76
	Rosa kwangtungensis	KP093152.1	91.52
	Rosa moschata subsp. *plena*	AB043005.1	91.43
	Rosa multiflora	AY635026.1	91.19
	Rosa odorata var. *erubescens*	FJ527705.1	90.90
	Rosa primula	FJ527717.1	90.76
	Rosa xanthina	FJ416655.1	90.90
	Rosa longicuspis	FJ416657.1	90.88
	Rosa multiflora var. *Carnea*	FJ527701.1	92.00
	Rosa soulieana	FM164950.1	95.00
	Rosa odorata var. *Gigantea*	HM593916.1	92.00
	Rosa odorata. var. *odorata*	HM593912.1	93.00
	Rosa davidii	FJ384670.1	90.62
	Rosa caudata	HM593912.1	93.00

	Rosa persetosa	FJ416658.1	89.96
	Rosa fedtschenkoana	MH712606.1	91.45
	Rosa davurica	FJ527710.1	90.36
	Rosa carolina	DQ242526.1	90.82
	Rosa virginiana	DQ242527.1	90.82
	Rosa blanda	MG237272.1	90.10
	Rosa rugosa	FJ527709.1	90.50
	Rosa woodsii	MG235846.1	90.00
	Rosa praelucens	FJ527708.1	90.63
	Rosa roxburghii	MH710748.1	91.04
	Rosa chinensis	FJ384671.1	90.93
	Rosa platyacantha	FJ527718.1	90.77
	Rosa stellata	AB048212.1	90.53
	Rosa banksiae	MH710729.1	90.00
	Rubus odoratus	KM037679.1	-
	Rubus bifrons	KM037668.1	-
*rbc*L	*Rosa arabica*	MT415957	-
	Rosa canina	FN689381.1	100.0
	Rosa chinensis	MH332770.1	99.65
	Rosa acicularis	MK714016.1	99.82
	Rosa bella	GQ436585.1	99.65
	Rosa blanda	KJ841522.1	99.47
	Rosa eglanteria	MK526526.1	99.55
	Rosa multiflora	NC_039989.1	99.65
	Rosa multiflora	MG893867.1	99.65
	Rosa glauca	KX163024.1	99.30
	Rosa gymnocarpa	KX678870.1	99.35
	Rosa hugonis	MG247629.1	98.75
	Rosa hugonis	MG247512.1	98.75
	Rosa kwangtungensis	KP094644.1	99.47
	Rosa laevigata	MN661139.1	99.65
	Rosa multiflora	NC_039989.1	99.65
	Rosa multiflora	MG893867.1	99.65
	Rosa nutkana	KY775761.1	99.47
	Rosa nutkana	MG248521.1	99.47
	Rosa palustris	HQ590246.1	99.67
	Rosa pisocarpa	MG249471.1	99.58
	Rosa pisocarpa	MG249038.1	99.50

	Rosa setigera	NK526528.1	98.47
	Rosa spinosissima	MK526539.1	98.55
*trn*L-F	*Rosa arabica*	MT427590	-
	Rosa multiflora	NC_039989.1	92.63
	Rosa multiflora	MG893867.1	92.63
	Rosa multiflora	MG727863.1	92.63
	Rosa canina subsp. *canina*	KF718324.1	92.72
	Rosa canina	MG387153.1	86.28
	Rosa eglanteria	KJ575176.1	82.53
	Rosa elliptica	KJ575131.1	86.15
	Rosa lasiosepala	KT359449.1	85.89
	Rosa pouzinii	KJ575171	86.79
	Rosa rubrifolia	KT359473.1	85.89
	Rosa rubus	GU575141.1	86.74
	Rosa sempervirens	KJ575180.1	86.30
	Rosa sherardii	KJ575188.1	86.12
	Rosa subcanina	KJ575194.1	86.30
	Rosa vosagiaca	KJ575203.1	86.30
*mat*K	*Rosa arabica*	MT416563	-
	Rosa canina	AB011980.1	99.93
	Rosa californica	AF288123.1	99.61
	Rosa eglanteria	JN895797.1	99.71
	Rosa multiflora var. *carnea*	HM490027.1	99.42
	Rosa multiflora	MG893867.1	99.51
	Rosa chinensis var. *spontanea*	NC_038102.1	99.51
	Rosa mollis	JN894862.1	99.63
	Rosa henryi	AB039310.1	99.63
	Rosa moschata	AB011990.1	99.39
	Rosa gallica	AB011978.1	99.51
	Rosa laevigata	MN372205.1	99.27
	Rosa roxburghii	FJ472517.1	99.27
	Rosa carolina	AB011983.1	99.49
	Rosa virginiana	AB011984.1	99.36
	Rosa woodsii	EU025926.1	99.27
	Rosa caudata	HM490015.1	99.71
	Rosa persetosa	HM490009.1	99.71
	Rosa xanthina	FJ472520.1	99.27

	Rosa davidii	HM490018.1	99.27
	Rosa praelucens	FJ472510.1	99.27
	Rosa banksiae	FJ472526.1	99.27
	Rosa sempervirens	AB048600.1	99.39
	Rosa setigera	AB048601.1	99.39
	Rosa spinosissima	AB039290.1	99.39
	Rosa gigantea	AB039314.1	99.27
	Rubus ulmifolius x *Rubus caesius*	KM036825.1	-

Table S2. Matrix of pairwise of ITS, *mat*K, *rbc*L, and *trn*L-F divergences for all samples studied.

Matrix of pairwise ITS divergences for all samples studied.

	R. arabica	*R. banksiae*	*R. blanda*	*R. canina*	*R. carolina*	*R. caudata*	*R. chinensis*	*R. davidii*	*R. davurica*	*R. dumalis* subsp. *dumalis*	*R. eglanteria*	*R. fedtschenkoana*	*R. gallica*	*R. henryi*	*R. kwangtungensis*	*R. laevigata*	*R. longicuspis*	*R. mollis*	*R. moschata*
R. arabica		80.20	82.70	81.90	83.10	80.20	81.50	81.20	82.30	81.90	82.50	83.40	82.50	82.40	83.20	82.50	81.10	83.60	82.80
R. banksiae	80.16		97.20	95.80	97.40	90.70	96.20	97.20	97.30	95.70	96.10	97.50	97.20	97.30	97.40	98.00	96.20	96.70	97.10
R. blanda	82.72	97.20		97.50	99.60	91.90	98.40	98.80	99.10	97.70	98.10	99.40	98.20	98.20	98.40	98.40	98.10	97.30	97.80
R. canina	81.93	95.80	97.50		97.70	91.60	98.10	97.50	97.60	98.50	99.40	97.90	97.50	97.80	98.00	98.30	97.30	97.30	97.50
R. carolina	83.07	97.40	99.60	97.70		92.10	98.60	99.00	99.30	97.90	98.30	99.60	98.40	98.50	98.60	98.60	98.30	97.50	98.00
R. caudata	80.22	90.70	91.90	91.60	92.10		91.80	91.80	92.00	91.40	91.80	92.50	91.20	91.40	92.00	91.40	91.40	91.90	91.50
R. chinensis	81.50	96.20	98.40	98.10	98.60	91.80		98.30	98.40	98.10	98.60	98.70	98.40	99.10	99.00	98.70	98.00	98.10	98.70
R. davidii	81.15	97.20	98.80	97.50	99.00	91.80	98.30		98.90	97.60	98.00	99.10	98.20	98.60	98.70	98.40	98.00	97.30	98.10
R. davurica	82.27	97.30	99.10	97.60	99.30	92.00	98.40	98.90		97.70	98.10	99.40	98.20	98.30	98.40	98.40	98.10	97.30	97.80
R. dumalis. subsp. *dumalis*	81.90	95.70	97.70	98.50	97.90	91.40	98.10	97.60	97.70		99.00	98.10	98.20	97.70	97.80	97.80	97.20	98.40	97.40
R. eglanteria	82.45	96.10	98.10	99.40	98.30	91.80	98.60	98.00	98.10	99.00		98.50	98.10	98.40	98.50	98.60	97.90	97.90	98.10
R. fedtschenkoana	83.38	97.50	99.40	97.90	99.60	92.50	98.70	99.10	99.40	98.10	98.50		98.50	98.50	98.70	98.70	98.40	97.60	98.10
R. gallica	82.53	97.20	98.20	97.50	98.40	91.20	98.40	98.20	98.20	98.20	98.10	98.50		98.40	98.40	98.70	98.10	97.60	98.20
R. henryi	82.36	97.30	98.20	97.80	98.50	91.40	99.10	98.60	98.30	97.70	98.40	98.50	98.40		99.60	98.70	98.40	97.60	99.60
R. kwangtungensis	83.24	97.40	98.40	98.00	98.60	92.00	99.00	98.70	98.40	97.80	98.50	98.70	98.40	99.60		98.80	98.50	97.80	99.10
R. laevigata	82.52	98.00	98.40	98.30	98.60	91.40	98.70	98.40	98.40	97.80	98.60	98.70	98.70	98.70	98.80		98.60	97.50	98.50
R. longicuspis	81.11	96.20	98.10	97.30	98.30	91.40	98.00	98.00	98.10	97.20	97.90	98.40	98.10	98.40	98.50	98.60		97.50	98.00
R. mollis	83.57	96.70	97.30	97.30	97.50	91.90	98.10	97.30	97.30	98.40	97.90	97.60	97.60	97.60	97.80	97.50	97.50		97.20
R. moschata	82.75	97.10	97.80	97.50	98.00	91.50	98.70	98.10	97.80	97.40	98.10	98.10	98.20	99.60	99.10	98.50	98.00	97.20	
R. moschata subsp. *plena*	83.78	97.30	98.40	98.10	98.60	92.00	99.00	98.70	98.40	97.90	98.70	98.70	98.40	99.60	99.70	98.80	98.50	97.80	99.10
R. multiflora	82.55	97.40	98.40	98.00	98.60	91.60	99.00	98.70	98.40	97.80	98.60	98.70	98.40	99.60	99.70	98.90	98.60	97.80	99.10
R. multiflora var. *carnea*	82.91	95.20	96.60	96.40	96.80	91.10	96.80	96.20	96.20	96.20	96.90	97.00	96.30	96.50	96.90	96.70	98.10	96.60	96.20
R. odorata var. *erubescens*	82.52	97.40	98.40	98.00	98.60	91.80	99.00	98.70	98.40	97.80	98.60	98.70	98.40	99.60	99.70	98.90	98.60	97.80	99.10
R. odorata var. *gigantea*	81.47	94.10	96.30	95.10	96.50	91.40	95.40	94.80	94.80	94.70	95.40	97.00	96.20	95.20	96.60	95.70	96.10	96.60	95.80
R. odorata var. *odorata*	81.63	96.70	97.40	97.60	97.60	91.30	98.00	97.40	97.40	97.10	97.80	97.60	97.40	97.70	97.80	98.10	98.70	96.80	97.20
R. persetosa	81.45	96.30	98.50	96.90	98.80	93.00	97.70	98.20	98.60	97.00	97.50	99.00	97.90	98.00	98.10	98.10	97.20	97.50	97.80

	R. moschata subsp. *plena*	*R. multiflora*	*R. multiflora* var. *carnea*	*R. odorata* var. *erubescens*	*R. odorata* var. *gigantea*	*R. odorata* var. *odorata*	*R. persetosa*	*R. phoenicia*	*R. platyacantha*	*R. praelucens*	*R. primula*	*R. roxburghii*	*R. rubiginosa*	*R. rugosa*	*R. sherardii*	*R. soulieana*	*R. stellata*	*R. virginiana*	*R. woodsii*	*R. xanthina*	*Rubus bifrons*	*Rubus odoratus*
R. arabica	83.80	82.60	82.90	82.50	81.50	81.60	81.50	82.30	82.40	82.30	82.30	82.40	96.30	82.10	90.70	82.00	83.80	83.10	82.50	81.60	71.27	74.90
R. banksiae	97.30	97.40	95.20	97.40	94.10	96.70	96.30	97.10	97.40	97.60	98.10	97.10	95.90	97.40	97.30	97.00	96.80	97.40	96.90	97.10	84.96	88.60
R. blanda	98.40	98.40	96.60	98.40	96.30	97.40	98.50	97.80	98.70	99.10	98.50	98.10	96.30	99.60	98.10	97.70	97.50	99.60	99.40	98.80	84.75	89.00
R. canina	98.10	98.00	96.40	98.00	95.10	97.60	96.90	97.70	98.00	97.80	98.10	98.00	96.30	97.70	98.20	97.80	97.00	97.70	97.20	98.20	84.40	88.60
R. carolina	98.60	98.60	96.80	98.60	96.50	97.60	98.80	98.00	98.90	99.30	98.80	98.30	96.30	99.80	98.20	97.90	97.70	99.90	99.30	99.00	84.82	89.10
R. caudata	92.00	91.60	91.10	91.80	91.40	91.30	93.00	91.40	91.70	92.30	91.80	91.10	95.90	92.00	97.30	91.30	91.40	92.10	91.70	92.10	79.25	83.60
R. chinensis	99.00	99.00	96.80	99.00	95.40	98.00	97.70	98.60	99.10	98.70	98.60	98.60	97.00	98.60	98.50	98.30	97.50	98.60	98.10	98.90	85.36	89.80
R. davidii	98.70	98.70	96.20	98.70	94.80	97.40	98.20	97.80	98.70	99.10	98.60	98.10	96.70	99.00	98.20	97.70	97.50	99.00	98.50	98.70	85.03	89.40
R. davurica	98.40	98.40	96.20	98.40	94.80	97.40	98.60	97.80	98.70	99.10	98.60	98.10	96.70	99.30	98.20	97.70	97.50	99.30	98.80	98.90	84.96	89.10
R. dumalis. subsp. *dumalis*	97.90	97.80	96.20	97.80	94.70	97.10	97.00	98.10	98.10	98.00	97.70	97.80	96.60	97.80	98.80	97.40	97.00	97.90	97.40	98.00	84.84	88.90
R. eglanteria	98.70	98.60	96.90	98.60	95.40	97.80	97.50	98.30	98.60	98.40	98.40	98.30	96.30	98.30	98.70	98.10	97.60	98.30	97.80	98.70	84.94	89.10
R. fedtschenkoana	98.70	98.70	97.00	98.70	97.00	97.60	99.00	98.10	99.10	99.40	98.80	98.40	96.70	99.60	98.30	97.90	97.80	99.60	99.10	99.10	84.99	89.00
R. gallica	98.40	98.40	96.30	98.40	96.20	97.40	97.90	97.80	98.70	98.50	98.50	98.10	98.60	98.40	99.00	97.70	97.20	98.40	97.90	98.80	84.88	88.80
R. henryi	99.60	99.60	96.50	99.60	95.20	97.70	98.00	98.10	99.00	98.60	98.60	98.40	96.60	98.40	98.30	98.00	97.40	98.50	97.90	98.80	84.91	89.20
R. kwangtungensis	99.70	99.70	96.90	99.70	96.60	97.80	98.10	98.20	99.10	98.70	98.70	98.50	97.00	98.50	98.70	98.10	97.60	98.60	98.10	99.00	84.84	89.20
R. laevigata	98.80	98.90	96.70	98.90	95.70	98.10	98.10	98.00	98.90	98.70	99.30	98.90	97.40	98.60	98.70	98.40	97.40	98.60	98.10	99.30	85.37	89.10
R. longicuspis	98.50	98.60	98.10	98.60	96.10	98.70	97.20	98.00	98.60	98.40	98.40	98.00	96.30	98.30	98.00	99.60	97.10	98.30	97.80	98.00	84.96	89.10
R. mollis	97.80	97.80	96.60	97.80	96.60	96.80	97.50	98.70	98.10	97.60	97.30	97.80	96.30	97.50	98.80	97.10	97.00	97.50	97.00	97.60	84.22	88.20
R. moschata	99.10	99.10	96.20	99.10	95.80	97.20	97.80	97.70	98.50	98.10	98.70	98.00	96.60	98.00	98.30	97.50	97.00	98.00	97.50	99.00	84.62	88.70
R. moschata subsp. *plena*		99.70	97.20	99.70	97.20	97.80	98.20	98.20	99.10	98.70	98.70	98.50	96.60	98.50	98.50	98.10	97.60	98.60	98.10	99.00	84.83	89.30
R. multiflora	99.70		96.70	99.70	95.30	97.90	98.10	98.30	99.10	98.70	98.70	98.60	97.00	98.60	98.50	98.10	97.60	98.60	98.10	99.00	84.96	89.30
R. multiflora var. *carnea*	97.20	96.70		96.70	96.80	96.80	95.90	96.10	96.70	96.50	96.50	96.10	96.30	96.40	98.00	97.70	96.40	96.80	96.30	96.80	83.19	87.50
R. odorata var. *erubescens*	99.70	99.70	96.70		95.30	97.80	98.10	98.30	99.10	98.70	98.70	98.60	97.00	98.60	98.70	98.10	97.60	98.60	98.10	99.00	85.08	89.40
R. odorata var. *gigantea*	97.20	95.30	96.80	95.30		97.10	95.00	94.70	95.30	95.10	95.40	95.00	95.50	95.00	97.50	96.00	96.20	96.50	96.00	95.40	81.83	85.80
R. odorata var. *odorata*	97.80	97.90	96.80	97.80	97.10		97.40	97.30	97.80	97.70	98.00	97.60	95.50	97.60	97.50	98.60	96.40	97.60	97.10	98.00	84.43	88.40
R. persetosa	98.20	98.10	95.90	98.10	95.00	97.40		97.70	98.40	99.00	98.60	97.80	95.90	98.70	98.00	97.40	97.30	98.80	98.20	98.40	84.77	88.90

R. phoenicia	82.25	97.10	97.80	97.70	98.00	91.40	98.60	97.80	97.80	98.10	98.30	98.10	97.80	98.10	98.20	98.00	98.00	98.70	97.70	98.20	98.30	96.10	98.30	94.70	97.30	97.70		98.60	98.10	97.80	98.30	96.60	98.00	98.20	97.60	97.30	98.00	97.50	98.10	85.08	89.00
R. platyacantha	82.39	97.40	98.70	98.00	98.90	91.70	99.10	98.70	98.70	98.10	98.60	99.10	98.70	99.00	99.10	98.90	98.60	98.10	98.50	99.10	99.10	96.70	99.10	95.30	97.80	98.40	98.60		99.00	98.70	98.90	97.40	98.90	98.50	98.10	97.60	98.90	98.40	99.00	85.37	89.40
R. praelucens	82.27	97.60	99.10	97.80	99.30	92.30	98.70	99.10	99.10	98.00	98.40	99.40	98.50	98.60	98.70	98.70	98.40	97.60	98.10	98.70	98.70	96.50	98.70	95.10	97.70	99.00	98.10	99.00		98.90	98.40	96.70	99.30	98.30	98.00	97.80	99.30	98.80	99.10	85.39	89.80
R. primula	82.27	98.10	98.50	98.10	98.80	91.80	98.60	98.60	98.60	97.70	98.40	98.80	98.50	98.60	98.70	99.30	98.40	97.30	98.70	98.70	98.70	96.50	98.70	95.40	98.00	98.60	97.80	98.70	98.90		98.40	96.60	98.70	98.30	98.30	97.60	98.80	98.20	99.70	85.39	89.40
R. roxburghii	82.41	97.10	98.10	98.00	98.30	91.10	98.60	98.10	98.10	97.80	98.30	98.40	98.10	98.40	98.50	98.90	98.00	97.80	98.00	98.50	98.60	96.10	98.60	95.00	97.60	97.80	98.30	98.90	98.40	98.40		96.60	98.30	98.70	97.80	97.50	98.30	97.80	98.40	84.81	89.00
R. rubiginosa	96.25	95.90	96.30	96.30	96.30	95.90	97.00	96.70	96.70	96.60	96.30	96.70	98.60	96.60	97.00	97.40	96.30	96.30	96.60	96.60	97.00	96.30	97.00	95.50	95.50	95.90	96.60	97.40	96.70	96.60	96.60		96.30	97.80	95.90	95.90	96.30	96.30	97.00	80.66	83.00
R. rugosa	82.13	97.40	99.60	97.70	99.80	92.00	98.60	99.00	99.30	97.80	98.30	99.60	98.40	98.40	98.50	98.60	98.30	97.50	98.00	98.50	98.60	96.40	98.60	95.00	97.60	98.70	98.00	98.90	99.30	98.70	98.30	96.30		98.20	97.90	97.60	99.80	99.30	99.00	85.10	89.30
R. sherardii	90.70	97.30	98.10	98.20	98.20	97.30	98.50	98.20	98.20	98.80	98.70	98.30	99.00	98.30	98.70	98.70	98.00	98.80	98.30	98.50	98.50	98.00	98.70	97.50	97.50	98.00	98.20	98.50	98.30	98.30	98.70	97.80	98.20		97.70	97.90	98.20	97.90	98.70	86.38	90.70
R. soulieana	82.04	97.00	97.70	97.80	97.90	91.30	98.30	97.70	97.70	97.40	98.10	97.90	97.70	98.00	98.10	98.40	99.60	97.10	97.50	98.10	98.10	97.70	98.10	96.00	98.60	97.40	97.60	98.10	98.00	98.30	97.80	95.90	97.90	97.70		96.70	97.90	97.40	98.30	84.86	89.30
R. stellata	83.80	96.80	97.50	97.00	97.70	91.40	97.50	97.50	97.50	97.00	97.60	97.80	97.20	97.40	97.60	97.40	97.10	97.00	97.00	97.60	97.60	96.40	97.60	96.20	96.40	97.30	97.30	97.60	97.80	97.60	97.50	95.90	97.60	97.90	96.70		97.70	97.20	97.90	84.49	88.40
R. virginiana	83.07	97.40	99.60	97.70	99.90	92.10	98.60	99.00	99.30	97.90	98.30	99.60	98.40	98.50	98.60	98.60	98.30	97.50	98.00	98.60	98.60	96.80	98.60	96.50	97.60	98.80	98.00	98.90	99.30	98.80	98.30	96.30	99.80	98.20	97.90	97.70		99.30	99.00	84.82	89.10
R. woodsii	82.51	96.90	99.40	97.20	99.30	91.70	98.10	98.50	98.80	97.40	97.80	99.10	97.90	97.90	98.10	98.10	97.80	97.00	97.50	98.10	98.10	96.30	98.10	96.00	97.10	98.20	97.50	98.40	98.80	98.20	97.80	96.30	99.30	97.90	97.40	97.20	99.30		98.50	84.75	88.90
R. xanthina	81.63	97.10	98.80	98.20	99.00	92.10	98.90	98.70	98.90	98.00	98.70	99.10	98.80	98.80	99.00	99.30	98.00	97.60	99.00	99.00	99.00	96.80	99.00	95.40	98.00	98.40	98.10	99.00	99.10	99.70	98.40	97.00	99.00	98.70	98.30	97.90	99.00	98.50		85.91	89.80
Rubus bifrons	71.27	85.00	84.80	84.40	84.80	79.30	85.40	85.00	85.00	84.80	84.90	85.00	84.90	84.90	84.80	85.40	85.00	84.20	84.60	84.80	85.00	83.20	85.10	81.80	84.40	84.80	85.10	85.40	85.40	85.40	84.80	80.70	85.10	86.40	84.90	84.50	84.80	84.80	85.90		90.60
Rubus odoratus	74.93	88.60	89.00	88.60	89.10	83.60	89.80	89.40	89.10	88.90	89.10	89.00	88.80	89.20	89.20	89.10	89.10	88.20	88.70	89.30	89.30	87.50	89.40	85.80	88.40	88.90	89.00	89.40	89.80	89.40	89.00	83.00	89.30	90.70	89.30	88.40	89.10	88.90	89.80	90.63	

Supplementary (Table S2.) Continued

Matrix of pairwise *mat*K divergences for all *Rosa* samples studied.

	R. arabica	*R. banksiae*	*R. californica*	*R. canina*	*R. carolina*	*R. caudata*	*R. chinensis*	*R. davidii*	*R. gallica*	*R. gigantea*	*R. henryi*	*R. laevigata*	*R. mollis*	*R. moschata*	*R. multiflora*	*R. multiflora* var. *carnea*	*R. persetosa*	*R. praelucens*	*R. roxburghii*	*R. sempervirens*	*R. setigera*	*R. spinosissima*	*R. virginiana*	*R. woodsii*	*R. xanthina*	*Rubus. ulmifolius*
R. arabica		99.33	99.21	99.70	99.33	99.33	99.57	99.33	99.57	99.33	99.45	99.33	99.69	99.45	99.57	99.57	99.33	99.33	99.33	99.45	99.45	99.45	99.33	99.33	99.33	95.79
R. banksiae	99.33		99.88	99.63	100	100	99.76	100	99.76	99.51	99.63	100	99.75	99.63	99.76	99.76	100	100	100	99.63	99.63	99.88	100	100	99.76	96.46
R. californica	99.21	99.88		99.51	99.88	99.88	99.63	99.88	99.63	99.39	99.51	99.88	99.63	99.51	99.63	99.63	99.88	99.88	99.88	99.51	99.51	99.76	99.88	99.88	99.63	96.46
R. canina	99.70	99.63	99.51		99.63	99.63	99.88	99.63	99.88	99.63	99.76	99.63	99.88	99.76	99.88	99.88	99.63	99.63	99.63	99.76	99.76	99.76	99.63	99.63	99.63	96.10
R. carolina	99.33	100	99.88	99.63		100	99.76	100	99.76	99.51	99.63	100	99.75	99.63	99.76	99.76	100	100	100	99.63	99.63	99.88	100	100	99.76	96.45
R. caudata	99.33	100	99.88	99.63	100		99.76	100	99.76	99.51	99.63	100.00	99.75	99.63	99.76	99.76	100.00	100.00	100.00	99.63	99.63	99.88	100.00	100.00	99.76	96.46
R. chinensis	99.57	99.76	99.63	99.88	99.76	99.76		99.76	100	99.76	99.88	99.76	100	99.88	100	100	99.76	99.76	99.76	99.88	99.88	99.63	99.76	99.76	99.51	96.22
R. davidii	99.33	100	99.88	99.63	100	100	99.76		99.76	99.51	99.63	100	99.75	99.63	99.76	99.76	100	100	100	99.63	99.63	99.88	100	100	99.76	96.46
R. gallica	99.57	99.76	99.63	99.88	99.76	99.76	100.00	99.76		99.76	99.88	99.76	100	99.88	100	100	99.76	99.76	99.76	99.88	99.88	99.63	99.76	99.76	99.51	96.22

R. gigantea	99.33	99.51	99.39	99.63	99.51	99.51	99.76	99.51	99.76		99.63	99.51	99.75	99.63	99.76	99.76	99.51	99.51	99.51	99.63	99.63	99.39	99.51	99.51	99.27	95.98
R. henryi	99.45	99.63	99.51	99.76	99.63	99.63	99.88	99.63	99.88	99.63		99.63	99.88	99.76	99.88	99.88	99.63	99.63	99.63	99.76	99.76	99.51	99.63	99.63	99.39	96.10
R. laevigata	99.33	100	99.88	99.63	100	100	99.76	100	99.76	99.51	99.63		99.75	99.63	99.76	99.76	100	100	100	99.63	99.63	99.88	100	100	99.76	96.46
R. mollis	99.69	99.75	99.63	99.88	99.75	99.75	100.00	99.75	100	99.75	99.88	99.75		99.88	100	100	99.75	99.75	99.75	99.88	99.88	99.63	99.75	99.75	99.51	96.20
R. moschata	99.45	99.63	99.51	99.76	99.63	99.63	99.88	99.63	99.88	99.63	99.76	99.63	99.88		99.88	99.88	99.63	99.63	99.63	99.76	99.76	99.51	99.63	99.63	99.39	96.34
R. multiflora	99.57	99.76	99.63	99.88	99.76	99.76	100.00	99.76	100	99.76	99.88	99.76	100	99.88		100	99.76	99.76	99.76	99.88	99.88	99.63	99.76	99.76	99.51	96.22
R. multiflora var. *carnea*	99.57	99.76	99.63	99.88	99.76	99.76	100.00	99.76	100	99.76	99.88	99.76	100	99.88	100.00		99.76	99.76	99.76	99.88	99.88	99.63	99.76	99.76	99.51	96.22
R. persetosa	99.33	100	99.88	99.63	100	100	99.76	100	99.76	99.51	99.63	100	99.75	99.63	99.76	99.76		100	100	99.63	99.63	99.88	100	100	99.76	96.46
R. praelucens	99.33	100	99.88	99.63	100	100	99.76	100	99.76	99.51	99.63	100	99.75	99.63	99.76	99.76	100.00		100	99.63	99.63	99.88	100	100	99.76	96.46
R. roxburghii	99.33	100	99.88	99.63	100	100	99.76	100	99.76	99.51	99.63	100	99.75	99.63	99.76	99.76	100	100		99.63	99.63	99.88	100	100	99.76	96.46
R. sempervirens	99.45	99.63	99.51	99.76	99.63	99.63	99.88	99.63	99.88	99.63	99.76	99.63	99.88	99.76	99.88	99.88	99.63	99.63	99.63		99.76	99.51	99.63	99.63	99.39	96.10
R. setigera	99.45	99.63	99.51	99.76	99.63	99.63	99.88	99.63	99.88	99.63	99.76	99.63	99.88	99.76	99.88	99.88	99.63	99.63	99.63	99.76		99.51	99.63	99.63	99.39	96.10
R. spinosissima	99.45	99.88	99.76	99.76	99.88	99.88	99.63	99.88	99.63	99.39	99.51	99.88	99.63	99.51	99.63	99.63	99.88	99.88	99.88	99.51	99.51		99.88	99.88	99.88	96.34
R. virginiana	99.33	100	99.88	99.63	100	100	99.76	100	99.76	99.51	99.63	100	99.75	99.63	99.76	99.76	100	100	100	99.63	99.63	99.88		100	99.76	96.46
R. woodsii	99.33	100	99.88	99.63	100	100	99.76	100	99.76	99.51	99.63	100	99.75	99.63	99.76	99.76	100	100	100	99.63	99.63	99.88	100		99.76	96.46
R. xanthina	99.33	99.76	99.63	99.63	99.76	99.76	99.51	99.76	99.51	99.27	99.39	99.76	99.51	99.39	99.51	99.51	99.76	99.76	99.76	99.39	99.39	99.88	99.76	99.76		96.22
Rubus ulmifolius	95.79	96.46	96.46	96.10	96.45	96.46	96.22	96.46	96.22	95.98	96.10	96.46	96.20	96.34	96.22	96.22	96.46	96.46	96.46	96.10	96.10	96.34	96.46	96.46	96.22	

Supplementary (Table S2.) Continued

Matrix of pairwise *rbc*L divergences for all *Rosa* samples studied.

	R. arabica	*R. acicularis*	*R. bella*	*R. blanda*	*R. canina*	*R. chinensis*	*R. rubiginosa*	*R. glauca*	*R. gymnocarpa*	*R. hugonis*	*R. hugonis 2*	*R. kwangtungensis*	*R. laevigata*	*R. multiflora*	*R. multiflora 2*	*R. nutkana*	*R. nutkana 2*	*R. palustris*	*R. pisocarpa*	*R. pisocarpa 2*	*R. setigera*	*R. spinosissima*	*R. tuberculatus*
R. arabica		99.82	98.62	98.43	97.97	98.62	97.45	97.3	99.64	99.46	99.82	99.47	98.62	99.64	99.64	97.47	99.64	98.6	99.64	99.64	99.64	99.82	96.62
R. acicularis	99.82		99.82	99.64	99.82	99.82	99.45	99.45	99.82	99.64	100	99.64	99.82	99.82	99.82	99.82	99.82	99.82	99.82	99.82	99.82	100	98.72
R. bella	98.62	99.82		99.84	99.43	99.72	99.14	99.48	100	99.64	99.82	99.64	99.57	99.82	99.82	99.15	100	99.84	100	100	99.82	99.82	98.62
R. blanda	98.43	99.64	99.84		99.51	99.67	98.95	99.3	99.82	99.46	99.64	99.46	99.67	99.64	99.64	98.96	99.82	99.67	99.82	99.82	99.64	99.64	98.43
R. canina	97.97	99.82	99.43	99.51		99.57	98.98	99.33	99.64	99.46	99.82	99.47	99.43	99.64	99.64	98.84	99.64	99.67	99.64	99.64	99.64	99.82	98.66
R. chinensis	98.62	99.82	99.72	99.67	99.57		99.31	99.65	99.82	99.82	99.82	99.82	99.86	100	100	98.97	99.82	100	99.82	99.82	100	99.82	98.62
R. rubiginosa	97.45	99.45	99.14	98.95	98.98	99.31		99.66	99.46	99.46	99.46	99.47	99.31	99.64	99.64	98.98	99.46	99.3	99.46	99.46	99.64	99.46	97.96
R. glauca	97.3	99.45	99.48	99.3	99.33	99.65	99.66		99.46	99.46	99.46	99.47	99.65	99.64	99.64	99.33	99.46	99.65	99.46	99.46	99.64	99.46	98.33
R. gymnocarpa	99.64	99.82	100	99.82	99.64	99.82	99.46	99.46		99.64	99.82	99.64	99.82	99.82	99.82	100	100	99.82	100	100	99.82	99.82	98.55
R. hugonis	99.46	99.64	99.64	99.46	99.46	99.82	99.46	99.46	99.64		99.64	99.64	99.82	99.82	99.82	99.64	99.64	99.82	99.64	99.64	99.82	99.64	98.37
R. hugonis2	99.82	100	99.82	99.64	99.82	99.82	99.46	99.46	99.82	99.64		99.64	99.82	99.82	99.82	99.82	99.82	99.82	99.82	99.82	99.82	100	98.73
R. kwangtungensis	99.47	99.64	99.64	99.46	99.47	99.82	99.47	99.47	99.64	99.64	99.64		99.82	99.82	99.82	99.64	99.64	99.82	99.64	99.64	99.82	99.64	98.4
R. laevigata	98.62	99.82	99.57	99.67	99.43	99.86	99.31	99.65	99.82	99.82	99.82	99.82		100	100	98.97	99.82	100	99.82	99.82	100	99.82	98.62
R. multiflora	99.64	99.82	99.82	99.64	99.64	100	99.64	99.64	99.82	99.82	99.82	99.82	100		100	99.82	99.82	100	99.82	99.82	100	99.82	98.55
R. multiflora2	99.64	99.82	99.82	99.64	99.64	100	99.64	99.64	99.82	99.82	99.82	99.82	100	100		99.82	99.82	100	99.82	99.82	100	99.82	98.55
R. nutkana	97.47	99.82	99.15	98.96	98.84	98.97	98.98	99.33	100	99.64	99.82	99.64	98.97	99.82	99.82		100	98.96	100	100	99.82	99.82	98.5
R. nutkana2	99.64	99.82	100	99.82	99.64	99.82	99.46	99.46	100	99.64	99.82	99.64	99.82	99.82	99.82	100		99.82	100	100	99.82	99.82	98.55
R. palustris	98.6	99.82	99.84	99.67	99.67	100	99.3	99.65	99.82	99.82	99.82	99.82	100	100	100	98.96	99.82		99.82	99.82	100	99.82	98.6
R. pisocarpa	99.64	99.82	100	99.82	99.64	99.82	99.46	99.46	100	99.64	99.82	99.64	99.82	99.82	99.82	100	100	99.82		100	99.82	99.82	98.55
R. pisocarpa2	99.64	99.82	100	99.82	99.64	99.82	99.46	99.46	100	99.64	99.82	99.64	99.82	99.82	99.82	100	100	99.82	100		99.82	99.82	98.55
R. setigera	99.64	99.82	99.82	99.64	99.64	100	99.64	99.64	99.82	99.82	99.82	99.82	100	100	100	99.82	99.82	100	99.82	99.82		99.82	98.55

R. spinosissima	99.82	100	99.82	99.64	99.82	99.82	99.46	99.46	99.82	99.64	100	99.64	99.82	99.82	99.82	99.82	99.82	99.82	99.82	99.82	99.82		98.73
R. tuberculatus	96.62	98.72	98.62	98.43	98.66	98.62	97.96	98.33	98.55	98.37	98.73	98.4	98.62	98.55	98.55	98.5	98.55	98.6	98.55	98.55	98.55	98.73	

Supplementary (Table S2.) Continued

Matrix of pairwise *trn*L-F divergences for all *Rosa* samples studied.

	R. arabica	*R. agrestis*	*R. arvensis*	*R. canina*	*R. canina 2*	*R. rubiginosa*	*R. elliptica*	*R. gallica*	*R. glauca*	*R. lasiosepala*	*R. multiflora*	*R. multiflora 2*	*R. pouzinii*	*R. rubrifolia*	*R. rubus*	*R. sempervirens*	*R. sherardii*	*R. subcanina*	*R. vosagiaca*	*Rubus KM037167*	*Rubus KM036980*
R. arabica		48.84	48.84	48.84	48.84	48.78	48.84	48.84	48.84	48.59	48.59	48.59	48.84	48.84	48.59	48.84	48.84	48.97	48.84	48.57	47.81
R. agrestis	48.84		100	100	100	99.54	100	100	100	99.71	99.71	99.71	100	100	99.71	100	100	99.86	100	84.73	84.73
R. arvensis	48.84	100		100	100	99.54	100	100	100	99.71	99.71	99.71	100	100	99.71	100	100	99.86	100	84.73	84.73
R. canina	48.84	100	100		100	99.54	100	100	100	99.71	99.71	99.71	100	100	99.71	100	100	99.86	100	84.73	84.73
R. canina 2	48.84	100	100	100		99.54	100	100	100	99.71	99.71	99.71	100	100	99.71	100	100	99.86	100	84.73	84.73
R. rubiginosa	48.78	99.54	99.54	99.54	99.54		99.54	99.54	99.54	99.54	99.54	99.54	99.54	99.54	99.54	99.54	99.54	99.54	99.54	86.22	86.22
R. elliptica	48.84	100	100	100	100	99.54		100	100	99.71	99.71	99.71	100	100	99.71	100	100	99.86	100	84.73	84.73
R. gallica	48.84	100	100	100	100	99.54	100		100	99.71	99.71	99.71	100	100	99.71	100	100	99.86	100	84.73	84.73
R. glauca	48.84	100	100	100	100	99.54	100	100		99.71	99.71	99.71	100	100	99.71	100	100	99.86	100	84.73	84.73
R. lasiosepala	48.59	99.71	99.71	99.71	99.71	99.54	99.71	99.71	99.71		100	100	99.71	99.71	100	99.71	99.71	99.57	99.71	84.73	84.73
R. multiflora	48.59	99.71	99.71	99.71	99.71	99.54	99.71	99.71	99.71	100		100	99.71	99.71	100	99.71	99.71	99.57	99.71	84.73	84.73
R. multiflora2	48.59	99.71	99.71	99.71	99.71	99.54	99.71	99.71	99.71	100	100		99.71	99.71	100	99.71	99.71	99.57	99.71	84.73	84.73
R. pouzinii	48.84	100	100	100	100	99.54	100	100	100	99.71	99.71	99.71		100	99.71	100	100	99.86	100	84.73	84.73
R. rubrifolia	48.84	100	100	100	100	99.54	100	100	100	99.71	99.71	99.71	100		99.71	100	100	99.86	100	84.73	84.73
R. rubus	48.59	99.71	99.71	99.71	99.71	99.54	99.71	99.71	99.71	100	100	100	99.71	99.71		99.71	99.71	99.57	99.71	84.73	84.73
R. sempervirens	48.84	100	100	100	100	99.54	100	100	100	99.71	99.71	99.71	100	100	99.71		100	99.86	100	84.73	84.73
R. sherardii	48.84	100	100	100	100	99.54	100	100	100	99.71	99.71	99.71	100	100	99.71	100		99.86	100	84.73	84.73
R. subcanina	48.97	99.86	99.86	99.86	99.86	99.54	99.86	99.86	99.86	99.57	99.57	99.57	99.86	99.86	99.57	99.86	99.86		99.86	84.58	84.58
R. vosagiaca	48.84	100	100	100	100	99.54	100	100	100	99.71	99.71	99.71	100	100	99.71	100	100	99.86		84.73	84.73
Rubus KM037167	48.57	84.73	84.73	84.73	84.73	86.22	84.73	84.73	84.73	84.73	84.73	84.73	84.73	84.73	84.73	84.73	84.73	84.58	84.73		97.61
Rubus KM036980	47.81	84.73	84.73	84.73	84.73	86.22	84.73	84.73	84.73	84.73	84.73	84.73	84.73	84.73	84.73	84.73	84.73	84.58	84.73	97.61	

Publisher's Note: MDPI stays neutral with regard to jurisdictional claims in published maps and institutional affiliations.

© 2020 by the authors. Submitted for possible open access publication under the terms and conditions of the Creative Commons Attribution (CC BY) license (http://creativecommons.org/licenses/by/4.0/).
